# Psychiatric genetic counseling for patients with schizophrenia and their families

**DOI:** 10.3389/fpsyt.2022.1014069

**Published:** 2022-10-10

**Authors:** Carla Kotzé, Gopolang Zwide

**Affiliations:** Department of Psychiatry, Faculty of Health Sciences, School of Medicine, Weskoppies Psychiatric Hospital, University of Pretoria, Pretoria, South Africa

**Keywords:** genetic, counseling, psychiatric, schizophrenia, environmental interaction

## Abstract

Steady progress in the field of psychiatric genetics, generating new and fascinating insight into the genetic and phenotypic complexity of schizophrenia and other serious mental illnesses, have created an increased need of psychiatric genetic counseling. It is a crucial aspect of psychiatric clinical practice to ensure a balanced approach that takes into consideration genetic and environmental risk factors and ongoing education in this rapidly developing field is essential. Genetic counseling will be increasingly important to assist patients with schizophrenia and their families to make a meaningful informed decision about genetic testing. It will preempt unrealistic expectations, discrimination and stigma related to incomplete understanding of genetic test results in the psychiatric context.

## Introduction

Major psychiatric disorders, including schizophrenia are complex, polygenic disorders. Genetic and environmental factors contribute to the susceptibility of an individual to develop schizophrenia (1). It is well-established that the risk of schizophrenia and many other mental illnesses runs in families with adoption, twin and family studies pointing to a heredity transmission of risk. It was expected that gene discovery will transform clinical psychiatric practice, but the genetics of mental illness have been found to be enormously complex (2).

The identification of genes that contribute to the risk of developing a mental disorder and influence the course of the illness is the goal of molecular genetic studies in the field. Vulnerability to a mental illness is a genetically complex trait that emerges from the interaction of multiple genes. This combination of genes do not fully control the risk of developing a mental illness and is dependent on the interaction with non-genetic factors (2). There has been steady progress in the field of psychiatric genetics, generating new and fascinating insight into the genetic and phenotypic complexity of mental illness (3).

Advances in psychiatric genetics has unraveled the genetic architecture of mental illness. The challenge psychiatrists face is the translation of the genetic findings into clinical practice to improve the lives of patients and families. Psychiatric genetic counseling (PGC) is a minor sub-specialty of genetic counseling that is not routinely provided for psychiatric conditions and there is a lack of consensus about the best way to deliver it to patients (4). It is expected that more people will seek PGC as technological and scientific advances enable more precise identification of individual genetic risks. Due to the complexity of psychiatric disorders PGC is among the most challenging topics encountered in clinical practice. Professional education is needed to better prepare psychiatrists to provide PGC in a manner that will assist patients and families to better understand the genetics of schizophrenia (5).

## Background

Psychiatrists regularly have to answer questions about heritability, genetic risks and the role of genetic testing in diagnosis or treatment selection (6). To better equip psychiatrists in training for future encounters where families or patients ask questions related to the genetics of schizophrenia, a postgraduate presentation was prepared as part of the academic program at the University of Pretoria. Based on this presentation, the aim of this perspective article is to summarize some of the important findings regarding the genetics of schizophrenia and to provide a brief guide for the general psychiatrist on implementation of PGC in clinical practice.

## Discussion

### Genetic influences in the development of schizophrenia

Specific details about the exact gene, receptor or neurotransmitter involved are not required during the process of PGC, but the information should be detailed enough for a patient or family to make an informed decision about genetic testing. Some of the important findings to take into consideration will be summarized here, without going into specific gene, receptor or molecular details. There has been a lot of progress in psychiatric genetics due to genome-wide association studies (GWAS) with the focus on identifying the underlying biological mechanisms contributing to disease. It includes investigation of polygenic mechanisms and examination of copy number variants (CNVs) and potential clinical applications for polygenic risk scores that calculate the joint contribution of common variants. The aim is to identify the genetic influences on mental disorders and rare single-nucleotide variants (SNVs) have been associated with schizophrenia and other psychiatric disorders (7).

It is important to explain clearly that CNVs and SNVs may be inherited or they may occur *de novo* and that CNVs predispose to a variety of psychiatric disorders with limited diagnostic specificity. CNVs tend to be expressed in the developing brain suggesting a common neurodevelopmental origin for many psychiatric disorders indicating that primary prevention would need to start in childhood (8, 9). An etiological overlap between major psychiatric disorders have been found with common genetic variations in neuronal, immune and histone pathways. The strongest GWAS finding specifically for schizophrenia was the association with genetic markers across the Major Histocompatibility Complex (MHC) locus on chromosome 6 and the C4 gene. This is hypothesized to cause an excess of synaptic pruning in the brain during the postnatal period (10).

Different approaches have been used to study common and rare genetic causes of schizophrenia and have contributed to advancement in the understanding of the biology of the disorder. There has been debate if the biological processes underlying schizophrenia is attributable mainly to common or rare Deoxyribonucleic Acid (DNA) variants and the evidence points to important roles for both. The tissue and cell types in which the newly identified risk variants are most biologically active has been found to be in brain tissue. On cellular level, neuronal cell subtypes have been most closely associated with the risk to develop schizophrenia (11). Individuals with schizophrenia have been found to have significantly higher rates of private damaging mutations in genes that are critical to synaptic function (12) and they also were found to have an excess of ultra-rare coding variants (UVRs) with the greatest expression in the central nervous system neurons with diverse molecular functions. The association with specific N-methyl-D-aspartate receptor subunits support the hypothesis that dysfunction of the glutamatergic system plays a role in the pathogenesis of schizophrenia (13). Rare disruptive coding variants influencing neuronal functioning processes, including differentiation, synaptic organization and transmission have been found in patients with schizophrenia. Schizophrenia is considered a complex brain disorder involving cortical and subcortical dysfunction with a spectrum of variations (14). The common variant associations at 287 genomic loci and 120 genes (106 protein-coding) identified by gene prioritization as likely to have causal roles in schizophrenia will be the focus of investigations for years to come to answer the profoundly complex challenges in our understanding of schizophrenia (15).

### Epigenetics and schizophrenia

Epigenetics is defined at the study of chemical changes at the cellular level that alter gene expression without altering the genetic code and is a significant consideration for PGC (6, 16, 17). It is now understood that the interaction between the individual and their environment can modify gene expression positively or negatively. Environmental factors such as stress, cigarette smoking, cannabis use and nutrition can influence the expression of genes and in turn cell activity and the consequent behavioral phenotypes. During PGC the psychosocial environment should be considered in combination with observed behavioral phenotypes. In patients with schizophrenia, adverse environmental influences or traumatic life experiences may contribute up to 60% to the development of the disorder. The flexibility of epigenetics and gene expression should be emphasized during PGC to engender hope, to address unfavorable influences and promote healthy environments and activities (16, 18).

### Patterns of inheritance and recurrence risk for schizophrenia

Like many mental disorders, schizophrenia have a robust genetic component reflected in the high degree of heritability. In the simplest terms, it can be explained to a family that if a family member has been diagnosed with major psychiatric disorder it increases other family members' risk of developing a similar disorder. There is an overlap in common genetic vulnerability markers between schizophrenia and other disorders, mostly bipolar disorder, that should be taken into consideration. Individuals can also have a higher risk to develop a mental disorder due to specific genetic mutations or polymorphisms without a family history (8, 9).

In patients with schizophrenia there will be one or more than one gene that interact with each other and interact with a variety of environmental factors, following a non-mendelian pattern of inheritance. The genetic heterogeneity means that the phenotype is not certain to be an indication of the genotype and several genetic mechanisms like mutation at separate loci or different mutations at a locus can lead to the same or similar phenotype (2). Multifactorial disorders that result from an interaction of one or more genes with one or more environmental factors are important considerations in PGC. In polygenic disorders such as schizophrenia the condition results from a cumulative effect of many genes acting independently to produce the overall effect (8).

The probability estimates of developing an illness may be useful for an individual or family members, but recurrence risk estimates for schizophrenia can be difficult and complex. The recurrence risk estimates are based on empiric risks and on statistical probabilities of particular study populations. The genetic risk will not translate to a specific family where the interaction with environmental factors may increase or lower the risk. The difficulty to predict the probability of schizophrenia developing in a specific family member, because of the complexity of multiple genes interacting with environmental risk factors should be addressed during PGC (2, 8). To determine the risk in a particular family, the number of affected family members might be used as an indication of a more highly genetic form of the disorder with a higher recurrence risk. Other factors that might influence the risk can include the sex of the affected family member, early onset of the disorder and the presence of specific pre-morbid traits such as early deviant behavior that might indicate a greater genetic loading (2, 19).

The genetic architecture of these disorders is becoming clearer and schizophrenia has been found to be 81% heritable with single nucleotide polymorphism (SNP) based estimates currently explaining 24% of heritability (8). The recurrence risk for schizophrenia based on the relationship to an affected individual can be summarized as 40–48% for an identical twin, 10–17% for a fraternal twin, 9% for a sibling, 6–13% for a parent, 13% for offspring and 2–5% for second or third-degree relative. The risk has also been found to be stronger in the case of early onset schizophrenia, defined as younger than 25 years of age in genetic studies (8, 20).

The polygenicity and genetic heterogeneity of schizophrenia, and how variants that influence illness may be widely distributed in the general population should be made clear during PGC. Several environmental factors act throughout life to influence the likelihood of the development of schizophrenia and the influence of genes and environmental factors combined will have to exceed a threshold for an individual to present with schizophrenia. The environmental influences are not causal factors and it can be grouped into early development, proximal factors and onset factors. Early development factors can include obstetric complications and advanced paternal age, while proximal factors refer to social adversity, migration or urbanicity experienced by the individual. The onset can be triggered by substance use, trauma or social adversity (21). Despite the tremendous advances in the genetics of mental illness, it has shown to be too complex to yield clinical guidelines or recommendations for the use of genetic testing for risk prediction or diagnostic purposes (10).

### Psychiatric genetic counseling

**Figure 1:**
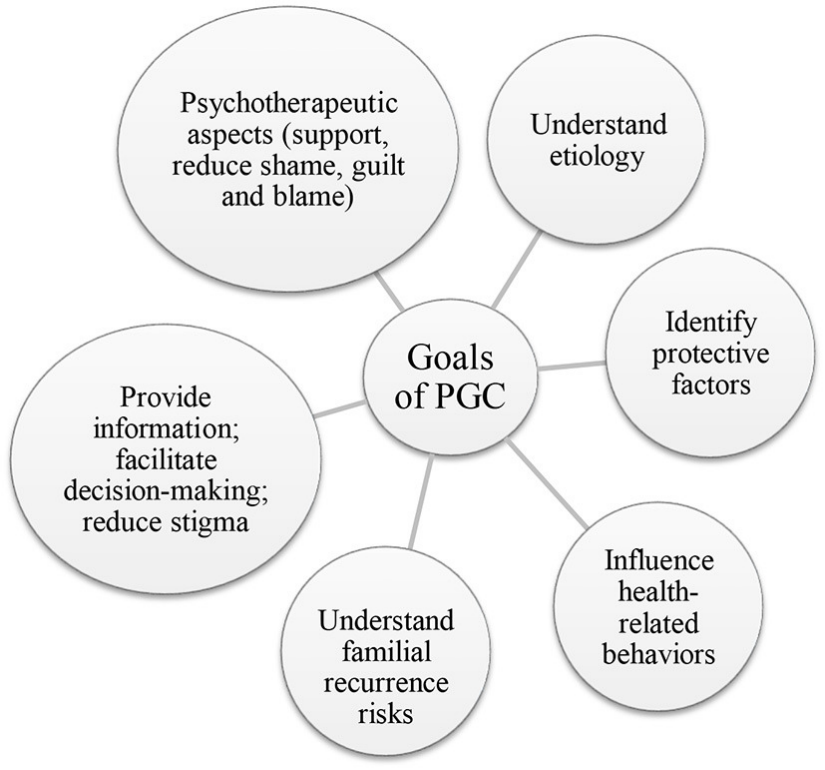
Goals and potential outcomes of PGC.

Genetic testing for schizophrenia has not been widely implemented and psychiatrists do not routinely use genetic testing as part of diagnostic practice and more training is needed in this field (22). The main aim of genetic testing is to develop more effective treatments and possibly prevent an illness from developing. At present, the clinical usefulness of pharmacogenomic profiles in patients with schizophrenia requires further investigation. Until there is sufficient scientific evidence to guide treatment choices, the majority of PGC will focus on the risk of mental illness in children and family members (10, 23). PGC might be useful for families where there is an individual who has been diagnosed with schizophrenia, including parents, siblings or spouses, people planning to adopt, and for the individual who has been diagnosed with schizophrenia. Explaining the technical issues of genetic testing is not enough for the purpose of PGC (24). The National Society of Genetic Counselors' Task force report from 2006 defined genetic counseling as the: “process of helping people understand and adapt to the medical, psychological, and familial implications of genetic contributions to disease.” (25). PGC should emphasize education, risk estimation and non-directive counseling to facilitate decision making (2). The goals of PGC are represented in Figure 1 and will be discussed in more detail below (4).

Understanding the etiology and familial recurrence risk: Genetic counseling in psychiatry should endeavor to help people understand the origin and role of genetics in psychiatric disorders (26). The limitations of risk prediction in genetically complex psychiatric disorders where there is an additive effect of multiple genes interacting with the environment, should be highlighted. Even if certain genes are identified, the diagnostic value may be limited and taking a good history, including a family history and doing a mental state examination will remain essential (2, 10). Currently, there are no genetic tests that can be used to confirm a diagnosis of schizophrenia, and a neurodevelopmental perspective with a focus on childhood development and early social and environmental influences will benefit clinical care (9, 26). The multifactorial model of inheritance and importance of gene-environment interactions should be explained during the provision of PGC in a manner that will be comprehensible to the patient and family. The context of the specific family's history can be used to explain complex concepts. This can be done by referring to affected and unaffected family members during the explanation of how the conditions might overlap, why some individuals might develop different conditions and some remain unaffected (25, 26). Understanding the individual and familial recurrence risk and precipitating factors can assist in decision-making about family planning and other important lifestyle choices to reduce risk and promote mental wellbeing in affected and unaffected individuals (27, 28).

Identifying protective factors and improving health-related behaviors: The discussions should also address how certain environmental factors, for example extreme stress, infections, malnutrition or substance use, amongst others, may trigger the onset, relapse or worsening of symptoms (28). PGC should provide the background for patients to identify areas where they can implement changes in their environment to address risks. General protective healthy behaviors such as effective ways to manage stress, adequate nutrition, sufficient sleep and regular exercise can be recommended, while advocating for avoidance of factors that can precipitate onset or relapse, such as substance use or stressful situations. These recommendations should be individualized according to the specific genetic and environmental risks of each individual and family involved and should also address the role of medication. The patients should be empowered to make informed decisions based on the best available evidence that may help prevent relapse or development of schizophrenia (29, 30).

Providing information to facilitate decision-making and to address stigma: The counselor should address potential harmful effects of genetic testing. These might include family conflicts, uncertainty about the probability of developing an illness and stigmatization. It can potentially influence important life decisions about a life partner and reproductive choices (31). Overreliance on a genetic explanation for schizophrenia can have very complex consequences on the stigma associated with the diagnosis (32). The advances in the field of genetics were initially seen as a potential way to address stigma, but an unintended consequence of an excessive focus on the neurobiological and genetic aspects may be increased feelings of despair with a sense of loss of control and permanence (33, 34). A focus on the abilities of the individual and integration into communities might address self-stigmatization more effectively, even without performing genetic testing (34). During PGC misconceptions must be explored and accurate information should be provided, including provision of information of about healthy lifestyle choices and behaviors that might enhance health. The aim is to strengthen the patient's sense of control over their disorder and to address feelings of guilt or self-stigmatization (26, 35).

Psychotherapeutic aspects: The process of PGC has been described extensively and consist broadly of information gathering (identifying needs, family history and details of the disease) and provision of information and support (36). The potential impact of genetic testing should be discussed before any decision to have testing done and the information provided should address the etiology, recurrence risk and decision-making support. During PGC there should also be support available after testing and assistance with the interpretation of the results (4, 25). The results should be explained in a non-judgmental and knowledgeable way. After the implications have been explained the patient should be empowered and should be offered personalized emotional support depending on the individual's needs (24). Genetic counseling is founded in a patient-centered approach and should combine educational and counseling components seamlessly. The counseling should address any specific genetic test result, but also interpersonal or other emotional issues that might arise to reduce feelings of shame, guilt and blame. The process of PGC will require firstly that information should be gathered and then explained to the patient in an understandable and supportive way. It should be a psychotherapeutic process that empowers the patient and family to implement lifestyle modifications that can address environmental risk factors (5, 23, 29). Genetic counseling can improve psychiatric care by provision of accurate information and clarification of misconceptions about the complex nature of psychiatric disorders and the equally complex and variable risk of recurrence (36, 37).

### Ethical considerations during PGC

There are many diverse and rapidly changing ethical concerns in psychiatric genetics. These concerns, especially in the field of research, big databases and direct-to-consumer-testing are vast. The brief discussion here will focus on some ethical aspects that should be considered during PGC in the clinical setting (32). The decision to have genetic testing done should remain autonomous and free by individuals with decision-making capacity. The decision should be based on the individual and cultural values of the person involved. To enhance clinical practice, values-based shared decision-making should be incorporated into the person-centered, evidence-based clinical care (24).

During the process of PGC issues of beneficence and non-maleficence should be considered. The potential harm should be balanced with the expected benefit from doing a specific genetic test and individuals should be supported to make a meaningful autonomous choice. These choices should be based on the individual values of the patient in the context of the family history, without any undue influence (23). The complexity of implications for patients and their families should not be underestimated and there may be a reasonable concern about discrimination based on genetic information. If the clinical significance of results for either prevention or treatment of schizophrenia is unclear, this should be explicitly stated (7, 8).

Whenever genetic tests are requested the implications for family members and the risk of incidental findings are important considerations. It should be discussed beforehand if an individual would want to learn of any incidental findings. As far as possible, families should participate in any decision that might affect them. Due to fast developments in the field of genomic medicine, continuing education should be combined with a dynamic process to provide information to enhance informed consent (7).

Misuse of genetic data to discriminate against individuals applying for insurance or in employment remain a concern (32). Concerns about discrimination, stigma, genetic essentialism and social use of genetic information are legitimate and loaded with ethical and political dilemmas. It is important to help patients and their families take all the information into consideration when they make decisions about genetic testing for schizophrenia or any other psychiatric disorder (7, 8).

## Conclusion

With the rapid and major developments in the field of genetics and genomics, it is important for psychiatrists to ensure that they remain up to date with the latest developments. PGC cannot be left only to specialized genetic counselors. It is a crucial aspect of psychiatric clinical practice to ensure a balanced approach that takes into consideration genetic and environmental risk factors. PGC will be increasingly important to assist patients to make a meaningful informed decision about genetic testing and will preempt unrealistic expectations, stigma and discrimination related to incomplete understanding of genetic test results in the psychiatric context.

## Data availability statement

The original contributions presented in the study are included in the article/supplementary material, further inquiries can be directed to the corresponding author/s.

## Author contributions

CK and GZ were involved with the original presentation that this perspective article is based on. All authors contributed to the write up of this perspective article.

## Conflict of interest

The authors declare that the research was conducted in the absence of any commercial or financial relationships that could be construed as a potential conflict of interest.

## Publisher's note

All claims expressed in this article are solely those of the authors and do not necessarily represent those of their affiliated organizations, or those of the publisher, the editors and the reviewers. Any product that may be evaluated in this article, or claim that may be made by its manufacturer, is not guaranteed or endorsed by the publisher.
